# Modeling of High-*T*_c_ Superconducting Bulk using Different *J*_c_–*T* Relationships over Dynamic Permanent Magnet Guideway

**DOI:** 10.3390/ma12182915

**Published:** 2019-09-09

**Authors:** Ye Hong, Jun Zheng, Hengpei Liao

**Affiliations:** Applied Superconductivity Laboratory, State Key Laboratory of Traction Power, Southwest Jiaotong University, Chengdu 610031, China

**Keywords:** high-temperature superconducting bulk, modeling, magnetic levitation, electromagnetic-thermo-force coupling, high speed

## Abstract

The linear temperature dependence of critical current density *J*_c_∝((*T*_c_*-T*)/(*T*_c_*-T*_0_)) and the nonlinear functions of *J*_c_∝(1-(*T*/*T*_c_)^2^)^α^ with the exponent α equal to 1, 3/2, and 2 are used to calculate the dynamic levitation force, the temperature distribution, and the current density distribution of the high-temperature superconducting (HTS) YBaCuO bulk over a permanent magnetic guideway (PMG). The calculations were based on the *H*-formulation and *E–J* power law. The model of the HTS bulk and the PMG has been built as a geometric entity by finite element software. To simulate the magnetic field fluctuation caused by the PMG arrangement irregularity, a small amplitude vibration in the vertical direction is applied to the PMG during the calculations. Both the low vibration frequency of 2 Hz and the high vibration frequency of 60 Hz are analyzed as the representative converted linear speeds of 34 km/h and 1018 km/h for magnetic levitation (Maglev) application. We compared the electromagnetic-thermo-force modeling with the experiments and the previous model without considering the thermal effect. The levitation force computed by the *J*_c_–*T* relationship, in which *J*_c_ is proportional to (1-(*T*/*T*_c_)^2^)^2^, is found to be in best agreement with the experimental data under quasi-static conditions. This work can provide a reference for the HTS electromagnetic-thermal-force coupling reproduction method of HTS Maglev at high speed.

## 1. Introduction

Due to the inherent flux-pinning effect, high-temperature superconducting (HTS) bulk has been potentially used in HTS magnetic levitation (Maglev) transportation [1,2,3,4] and many other applications [5,6]. To investigate the electromagnetic characteristic of the HTS Maglev system, a proper HTS *E*-*J* constitutive law is necessary. For the HTS materials, the critical current density changes along the temperature. In the past, Matsushita et al. [7] investigated the single-grain YBaCuO specimen by measuring the critical current density *J*_c_–*T*, and proposed the current density as a function of *1*-(*T*/*T*_c_)^2^. Yamasaki et al. [8] reported that *J*_c_ is proportional to *(1-AT +BT*^2^*),* where *A* and *B* were constants for the HTS material of Bi-2223 thin films. Another research study [9] described *J_c_* as a function of (*1-T*/*T*_c_)^γ^ based on the exploration of Bi-2212/Ag wires. In addition, *J*_c_∝(*1-*(*T*/*T*_c_)^2^)^α^ for Bi-2212 tapes was mentioned in the book [10]. For 2G HTS YBCO bulks, Braeck [11] assumed a linear temperature dependence of the critical current as *J*_c_∝((*T*_c_-*T*)/(*T*_c_-*T*_0_)); and then Tsuchimoto [12] presented an exact nonlinear relationship of *J*_c_∝(1-(*T*/*T*_c_)^2^)^2^. 

Later, from the point of view of superconducting YBaCuO application, Tsukamoto et al. [13] employed the linear temperature dependence of the critical current density to calculate the temperature variation and the trapped magnetic field in YBCO bulks under an AC external field. Using this linear formula, Tixador et al. [14] calculated the current distribution and AC losses of the YBCO slab. In recent years, Ye [15] and Huang [16] studied the dynamic thermal effect of the HTS Maglev system using YBaCuO bulks by the linear *J*_c_*–T* relation.

In this paper, in order to elucidate which *J_c_*–*T* relationship is more appropriate to model the HTS Maglev system, we used four different *J*_c_–*T* functions to calculate the dynamic levitation force, the temperature distribution, and the current density distribution of the HTS bulk over the applied permanent magnetic guideway (PMG). The modeling characterized by the *H*-formulation and the *E*–*J* power law was implemented in the finite element software COMSOL Multiphysics 5.3a. With the magnetic field formulation (MFH) interface in the AC/DC module, the calculation subdomains of the HTS bulk and the PMG were built as the geometric entity. During the calculations, a vertical vibration with small amplitude was applied to the PMG to simulate the magnetic field fluctuation caused by the inevitable PMG irregularity. Different from the previous modeling [17,18], the thermal effect was taken into account by coupling the heat transfer module. To study the levitation performance of the HTS Maglev system at high speed, a vibration with the frequency of 60 Hz was set to simulate the magnetic field inhomogeneity, which is equivalent to the linear velocity of about 1018 km/h of the circular PMG employed in the experiments. The converted linear velocity ***v*** of the circumferential PMG in the experiments can be expressed as:

(1) $$v\;\left(\mathrm{km}/h\right)=r\left(m\right)\times 2\pi n\left(\mathrm{rpm}\right)\times \frac{60}{1000}$$

where *r* is the radius of the circular PMG, which is 0.75 m; the rotation rate of the PMG, *n* (rpm), is derived from the vibration frequency of the guideway set in the simulation. This occurs since one cycle of the magnetic field fluctuation during the dynamic modeling is approximately tantamount to the real PMG magnetic field, which the HTS bulk subjects to when the circular guideway rotates for one circle in the experiment [19,20]. Thus, the cycle of the magnetic field fluctuation is 1/60 s when the frequency is 60 Hz, and the PMG’s rotation rate *n* is 3600 rpm. In that case, the calculation results by different *J*_c_–*T* functions showed the levitation force attenuation during the vibration process. In comparison with the measurements in this paper, the *J*_c_–*T* relationship in which *J*_c_ is proportional to (1-(*T*/*T*_c_)^2^)^2^ shows better agreement with the experiment.

## 2. Theoretical Model

The HTS bulk and the opposite -polar-arranged PMG were built as the geometric entity in the COMSOL. The remanence of the permanent magnet *B*_r_ was set at 0.8 T. With the MFH (magnetic field formulation) interface, we can easily simulate the magnetic field produced by any complex- shaped magnets. In this study, after the PMG reached the working height (WH), a small amplitude vertical vibration was applied to the PMG to simulate the magnetic field fluctuation. The first movement stage is regarded as the quasi-static process, since the speed is as low as 1 mm/s, while the following vibration process is the dynamic levitation stage.

**Figure 1:**
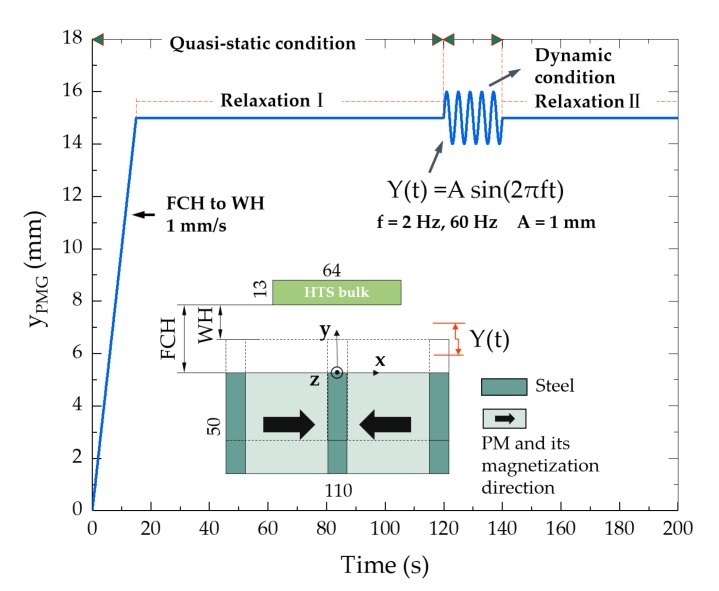
Vertical displacement variation of the permanent magnetic guideway (PMG) and two-dimensional (2D) model for the high-temperature superconducting (HTS) Maglev system. Vibration amplitude A = 1 mm. Field-cooling height (FCH) = 30 mm, working height (WH) = 15 mm. The quasi-static condition from 0 s to 120 s includes the 0–15 s upward movement from FCH to WH and the Relaxation I process from 15 to 120 s. Then, after 20 s of vibration, the second relaxation process (Relaxation II) happened from 140 s to the end.

Figure 1 shows the computation subdomains of the dynamic model and the movement process of the PMG. In the modeling, the PMG first moves from the field-cooling height (FCH = 30 mm) to the working height (WH = 15 mm) at 1 mm/s. This process takes 15 s. Afterwards, it relaxed from 15 to 120 s (Relaxation I) to let the flux fully redistribute. Then, a sine function with 1-mm amplitude is applied to the PMG for 20 s. After the dynamic condition, the second relaxation process (Relaxation II) takes place, which is from 140 s to the end. In the dynamic condition, the vibration frequency conditions of 2 and 60 Hz are calculated, respectively. According to the size of the circular PMG in the experimental equipment SCML-03 [21], the vibration frequencies of 2 and 60 Hz are corresponding to the linear velocity of 34 and 1018 km/h for the Maglev above the PMG, respectively. 

### Equations

To analyze the electromagnetic characteristics of the HTS bulk, the *H*-formulation and the well-known Maxwell equations (neglecting the displacement current term) [22] are as follows:

(2) $$\mu \frac{\partial H}{\partial t}+\;\nabla \;\times E=0$$

(3) E=ρ J

(4) J=∇×H

where *H* is the magnetic field strength; ρ is the resistivity of the material, which is assumed to be isotropic in the two-dimensional (2D) model; *E* is the electric field; and *J* is the current density. Equations (2)–(4) are solved by the MFH interface in the AC/DC module.

The strong nonlinear relationship between ***E*** and ***J*** of the HTS material can be characterized by the experimental empirical *E*–*J* power law [23]:

(5) $$E=E_{c}\;{\left(\frac{J}{J_{c}}\right)}^{m}$$

where *E*_c_ is the critical current criterion equal to 100 μV/m, and *m* is the power law exponent, which is usually 21. The parameter *J*_c_ in the constitutive law depends on the temperature *T*. Thus, the thermal effect is coupled in the modeling using the heat transfer module. Four *J*_c_–*T* relationships are considered. 

(1). The linear dependence [15]:

(6) $$J_{c}=J_{c0}\;\frac{T_{c}-T}{T_{c}-T_{0}}\;$$

where *J*_c0_ is the critical current density of the HTS bulk at *T* = *T*_0_ (77 K); *T*_c_ is the critical temperature; and *T*_0_ is the coolant temperature.

(2). The nonlinear dependence with α = 1, 3/2, 2 turns into [10]:

(7) $$J_{c}=J_{c1}{\left(1-{\left(\frac{T}{T_{c}}\right)}^{2}\right)}^{\alpha }\;$$

where the critical current density *J*_c1_ is obtained by Equation (6) with *T* = 0 K. In addition, we calculated the case without considering the thermal effects. In that case, *J*_c_ as a constant equals *J*_c0_.

The thermal equilibrium equation is expressed as:

(8) $$C_{p}\frac{\partial T}{\partial t}-\nabla \cdot \left(\lambda \nabla T\right)=EJ$$

where *C*_p_ is the heat capacity per unit volume of the superconductor; and *λ* is the thermal conductivity of the superconductor.

The levitation force is obtained by the Lorenz force formula at each time instant as:

(9) $$F_{y}\;\left(t\right)=\int_{S}\;B\times J\;dS\;\left[\frac{N}{m}\right]$$

where *S* is the cross-section of the HTS bulk in the *x-y* plane. We assume four bulks placed along the *z*-direction to get the larger force; the total levitation force equals *F_y_* (in the actual calculation, *F_y_* is the force density along the length at the *z*-direction) times 128 mm, since each HTS bulk is 32-mm wide along the *z*-direction and there are four bulks in the experiments. Table 1 summarizes the simulation parameters. The material properties of the HTS bulk are based on the melt textured three-seeded rectangular YBaCuO bulk made by the ATZ GmbH (Torgau, Germany).

## 3. Results

**Figure 2:**
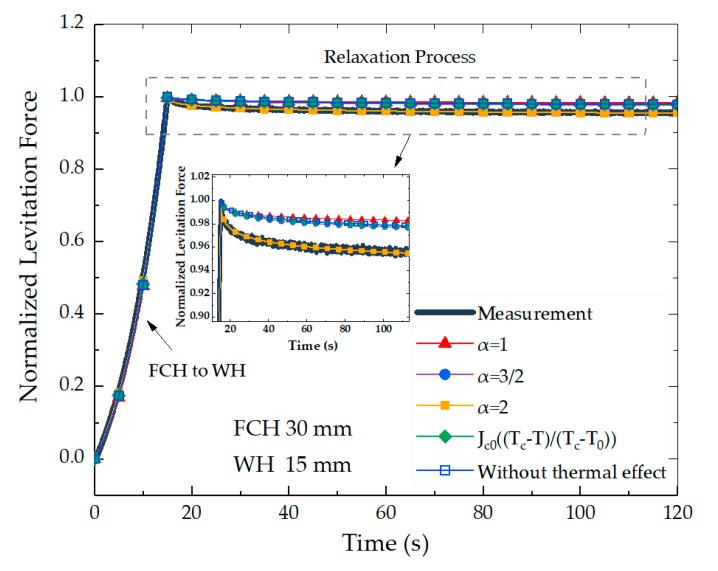
Normalized quasi-static levitation force versus time under different *J*_c_–*T* relationships of: *J*_c_ = *J*_c1_(*1*-(*T*/*T*_c_)^2^)*^α^*, α = 1, 3/2, 2; *J*_c_ = *J*_c0_((*T*_c_-*T*)/(*T*_c_-*T*_0_)); *J*_c_ = *J*_c0_. The PMG in the simulation (or bulks in the experiment) moved from the FCH (30 mm) to the WH (15 mm) at the vertical 1 mm/s speed, and then relaxed for over 100 s. The original values of the levitation force at 15 s were 126.40 N (measured), 224.40 N (α = 1), 216.58 N (α = 3/2), 203.62 N (α = 2), 216.52 N (*J*_c0_((*T*_c_-*T*)/(*T*_c_-*T*_0_)), and 216.51 N (*J*_c_ = *J*_c0_), respectively.

Figure 2 displays the normalized quasi-static levitation force obtained by calculation and experiment. In the experiment, four rectangular three-seeded YBaCuO bulks, fabricated by ATZ GmbH in Germany, were mounted in a sample holder fixed above the circular PMG of the SCML-03. The bulk size is 64 × 32 × 13 mm^3^. The cross-section of the PMG is the same as the geometry in the simulation, which is shown in Figure 1. The experiment process was the same as the quasi-static process of Figure 1. The YBaCuO bulks were first field cooled with a height of 30 mm, which is the distance between the top surface of the PMG and the bottom of the YBaCuO bulks. Afterwards, the bulks were brought down at 1 mm/s from the FCH to the WH (15 mm), and then relaxed for 10 min.

The numerical results are computed based on the *J*_c_–*T* functions of Equations (6) and (7) with α = 1, 3/2, and 2, as well as the case without considering thermal effect. The levitation forces obtained by calculation and measurement are normalized by dividing its maximum force at 15 s. The original values of the levitation force at 15 s were 126.40 N (measured), 224.40 N (α = 1), 216.58 N (α = 3/2), 203.62 N (α = 2), 216.52 N (*J*_c0_(*(T*_c_*-T*)/(*T*_c_*-T_0_*)), and 216.51 N (*J*_c_ = *J*_c0_), respectively. So, all the normalized levitation forces equaled 1 only at 15 s, to better compare the difference between the force trends. 

It is noted that the levitation force increases gradually during the PMG moving from the field-cooling height to the working height. Then, it shows clear attenuation at the first few seconds during the relaxation process. This is due to the great change of the external magnetic field caused by the large-range movement of the PMG or the HTS bulk, which leads to the redistribution of the flux inside the bulk. The inset in Figure 2 is the partial enlargement of the maximum force region. From the inset, we can see that the calculated levitation force with α equals 2 by Equation (7), which agrees best with the measurements in the quasi-static condition. 

**Figure 3:**
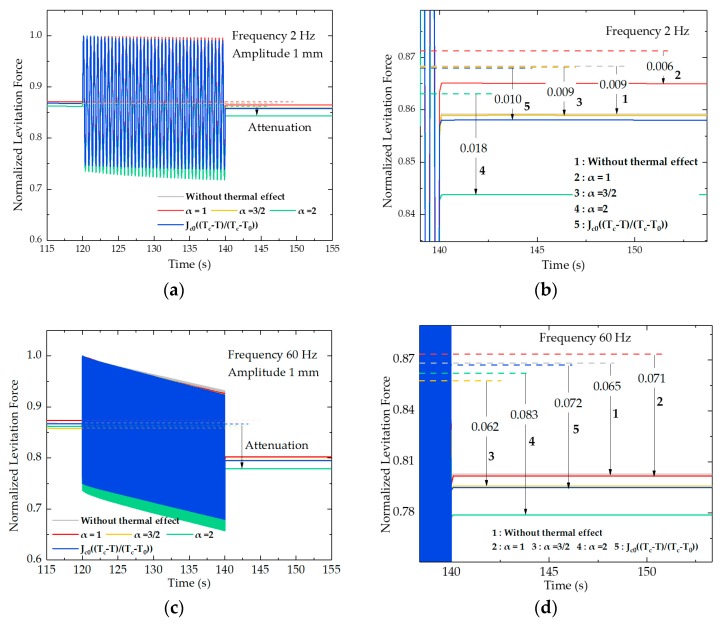
Normalized levitation force profiles by different *J*_c_–*T* relationships of *J*_c_ = *J*_c0_*; J*_c_ *= J*_c1_(*1-*(*T*/*T*_c_)^2^)*^α^*, α = 1, 3/2, 2; *J*_c_ *= J*_c0_((*T*_c_*-T*)/(*T*_c_*-T*_0_)) under (**a**) 2 Hz and (**c**) 60 Hz; (**b**) and (**d**) emphasize displaying the force attenuation caused by the vibration process under 2 Hz and 60 Hz, respectively. The dotted lines represent the force value at the end of Relaxation I (120 s). FCH = 30 mm, WH = 15 mm. 1, 2, 3, 4, and 5 represent five *J_c_–T* calculation conditions.

Figure 3 further displays the normalized dynamic levitation forces from 115 to 155 s at 2 and 60 Hz by five different calculations of different *J_c_–T* formulas. Figure 3a,c show the general view of the dynamic process, while Figure 3b,d zoom in the vibration end to better compare the different levitation force changes. The corresponding movement of the PMG is depicted in Figure 1. It is found that the continuous vibration of the PMG can lead to the levitation force attenuation. Table 2 collects the attenuation values of each case. The attenuation is the difference between the results obtained at the beginning (120 s) and the end (140 s) of the dynamic condition process, which can be seen in Figure 3b,d. We can find in Table 2 that with the increase of the frequency, the attenuation gets more obvious. This is because the flux inside the HTS bulk under higher vibration frequency is more intense. Other studies in the literature [15,16] have concluded that the levitation force is a little higher when not considering the thermal effect than when accounting for the thermal effect, because the attenuation caused by thermal loss is not considered. From this point of view, under the experimental vibration condition, the force attenuation without the thermal effect is a little smaller than the case considering the thermal effect, in which *J_c_* retains some mathematic relationships with temperature *T* during the calculations. As shown in Table 2, the calculated force attenuation without considering the thermal effect is 0.009 at 2 Hz, and 0.065 at 60 Hz. However, the calculation results considering the thermal effect, such as 0.006 under α = 1 at 2 Hz and 0.062 under α = 3/2 at 60 Hz, are smaller. In addition, 0.010 under the linear function and 0.009 under α = 3/2 at 2 Hz are almost the same, with 0.009 at 2 Hz without any thermal effect. Therefore, only the results calculated by Equation (7) with α = 2 are satisfied and reasonable, which is the same conclusion by the quasi-static comparison in Figure 2. 

**Figure 4:**
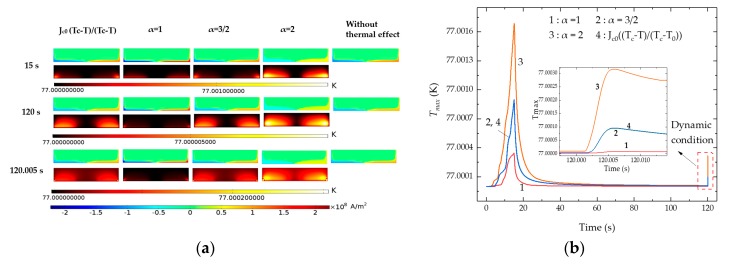
Comparison of different *J*_c_–*T* relationships of *J*_c_ *= J*_c1_(*1*-(*T*/*T*_c_)^2^)*^α^* with α = 1, 3/2, 2 and *J*_c_ *= J*_c0_((*T*_c_*-T*)/(*T*_c_*-T*_0_)) under 60 Hz. (**a**) Current density and temperature profile inside the HTS bulk at 15 s, 120 s, and 120.005 s; (**b**) maximum temperature *T*_max_ inside the HTS bulk versus time.

The current density and the temperature distribution of the HTS bulk under 60 Hz at the working height (15 s), the end of the first relaxation process (120 s), and the time when the PMG first reached the vibration peak (120.005 s) are shown in Figure 4a. It is seen that all the currents and the temperature rise appear from the bottom of the bulk, and the temperature gradually spreads to the entire bulk. All the modeling results indicate that the maximum temperature occurs at 15 s, when the PMG arrives at the working height after the quasi-static process. While the thermal effect by that tiny-amplitude vibration is small, it is concluded that compared to the small-amplitude high-frequency vibration of the PMG, a long-distance movement between the FCH and the WH is more likely to lead to temperature rise, because it causes a distinct magnetic field variation despite the speed being only 1 mm/s. Figure 4b displays the maximum temperature inside the HTS bulk calculated by different *J*_c_–*T* relationships under 60 Hz. It is seen that temperature rise occurs mainly around the time when the PMG first reached the working height, and the first upward moved 1 mm during the dynamic process. The inset in Figure 4b further indicates the temperature variation difference after the beginning of the vibration. Although the vibration process excites the second temperature rise, the temperature rise is much smaller than that in the first movement from the 30-mm FCH to the 15-mm WH, because the vibration amplitude is only 1 mm. Furthermore, the temperature curves kept decreasing gradually after the PMG first reached the peak position during dynamic conditions. 

## 4. Conclusions

In this study, based on the verification of the model by the measured levitation force, we calculated the levitation force, the temperature distribution, and the current density distribution of the HTS bulk under dynamic conditions. Four functions describing the temperature dependence of the critical current density (*J*_c_–*T*) were used in the calculations. The results were compared with the simulation case, without accounting for the thermal effect as well as the measurements. Considering the computation efficiency, we calculate 20 s for the dynamic operation, since the present computational condition is not capable of guaranteeing the long-time vibration calculation. The following conclusions are obtained:

1. The *J*_c_–*T* function of *J*_c_ proportional to *J*_c1_(*1*-(*T*/*T*_c_)^2^)^2^ is more appropriate to reproduce the electromagnetic-thermo-force coupling characteristics of the HTS Maglev system.
2. According to the calculated dynamic levitation force, it is predicted that the simulated dynamic levitation force running at a high speed such as 1018 km/h would decrease 8.3% at the beginning, if the applied PMG field could be inhomogeneous along the running direction, similar to the particularly designed inhomogeneous PMG in the SCML-03 system. On the other hand, it is another significant research issue to design or optimize the working magnetic field homogeneity of the PMG for the high-speed HTS Maglev application.

## Figures and Tables

**Table 1:** Parameters for the modeling.

Symbol	Value	Name
***E*_c_**	1 × 10^−4^ V/m	Critical current criterion
***J*_c0_**	1.1 × 10^8^ A/m^2^	Critical current density
***m***	21	Power law exponent
***B*_r_**	0.8 T	Remanence of the PM
***T*_c_**	92 K	Critical temperature
***T*_0_**	77 K	Initial temperature
***C*_p_**	132 J/(kg·K)	Heat capacity per unit volume
**λ**	4 W/(m·K)	Thermal conductivity

**Table 2:** The normalized levitation force attenuation under different ***J*_c_**–***T*** relationships.

Frequency	Converted Speed	*J*_c0_((*T_c_*-*T*)/(*T_c_*-*T*_0_))	*J*_c_*=J*_c1_(*1*-(*T*/*T*_c_)^2^)*^α^*			Without Thermal Effect
			α = 1	α = 3/2	α = 2	
*f* = 2 Hz	34 km/h	0.010	0.006	0.009	0.018	0.009
*f* = 60 Hz	1018 km/h	0.072	0.071	0.062	0.083	0.065
